# What are the trends in seeking health care for fever in children under-five in Sierra Leone? evidence from four population-based studies before and after the free health care initiative

**DOI:** 10.1371/journal.pone.0263364

**Published:** 2022-02-02

**Authors:** Joel Dofinissery Bognini, Sekou Samadoulougou, Mady Ouedraogo, Francis Smart, David Tiga Kankoye, Osman Sankoh, Fati Kirakoya-Samadoulougou

**Affiliations:** 1 Unité de Recherche Clinique de Nanoro, Institut de Recherche en Sciences de la Santé, Centre National de la Recherche Scientifique et Technologique, Ouagadougou, Burkina Faso; 2 Centre for Research on Planning and Development (CRAD), Laval University, Quebec, Canada; 3 Evaluation Platform on Obesity Prevention, Quebec Heart and Lung Institute, Quebec, Canada; 4 Institut National de la Statistique et de la Démographie [INSD], Ouagadougou, Burkina Faso; 5 Ministry of Health and Sanitation, Freetown, Sierra Leone; 6 Centre National de Recherche et de Formation sur le Paludisme (CNRFP), Ouagadougou, Burkina Faso; 7 Statistics Sierra Leone, Freetown, Sierra Leone; 8 School of Public Health, Faculty of Health Sciences, University of the Witwatersrand, Johannesburg, South Africa; 9 Centre de Recherche en Epidémiologie, Biostatistiques et Recherche Clinique, École de Santé Publique, Université Libre de Bruxelles (ULB), Bruxelles, Belgique; Njala University, SIERRA LEONE

## Abstract

**Background:**

In 2010, the government of Sierra Leone implemented the Free Health Care Initiative (FHCI) in the country with the objective of reducing the high maternal, infant, and child mortality rates and improving general health indicators. The objective of this study was to assess the trends in the prevalence of health care-seeking and to identify the determinants of healthcare service utilization by caregivers of children younger than five years.

**Methods:**

The analysis of health-care-seeking behavior was done using data from four population-based surveys in Sierra Leone before (2008) and after (2013, 2016, 2019) the FHCI was implemented. Care-seeking behavior was assessed with regard to caregivers seeking care for children under-five in the two weeks prior to each survey. We compared the percentages of healthcare-seeking behavior change and identify factors associated with healthcare-seeking using a modified Poisson regression model with generalized estimating equations.

**Results:**

In 2008, a total of 1208 children with fever were recorded, compared with 2823 children in 2013, 1633 in 2016, and 1464 in 2019. Care-seeking for children with fever was lowest in 2008 (51%; 95% CI (46.4−55.5)) than in 2013 (71.5%; 95% CI (68.4−74.5)), 2016 (70.3%; 95% CI (66.6−73.8)), and 2019 (74.6%; 95% CI (71.6−77.3)) (p < 0.001). Care-seeking in 2013, 2016 and 2019 was at least 1.4 time higher than in 2008 (p < 0.001) after adjusting for mother’s age, wealth, religion, education level, household head and the child’s age. Care-seeking was lowest for children older than 12 months, mothers older than 35 years, children living in the poorest households, and in the northern region. A trend was observed for the sex of the household head. The level of care-seeking was lowest when the household head was a man.

**Conclusions:**

The increase in healthcare-seeking for children under-five with fever followed the introduction of the FHCI in Sierra Leone. Care-seeking for fever varied by the child’s age, caregiver’s age, household wealth, the sex of the household head and region. Maintaining the FHCI with adequate strategies to address other barriers beyond financial ones is essential to reduce disparities between age groups, regions and, households.

## Background

Substantial progress was made in the control of the global under-five mortality rate (U5MR), with a 49% decline achieved in 18 years, from 76 per 1000 live births in 2000 to 39 per 1000 live births in 2018. The biggest share of mortality (41.3%) in this group is attributable to infectious diseases worsened by malnutrition [1] among which the leading killers are preventable and treatable febrile diseases [2]: respiratory diseases (12%), diarrheal diseases (8.3%), sepsis (7%), and malaria (5%). These diseases in children under the age of five remain a public health problem and hinder the optimal growth of children in low income countries, particularly in sub-Saharan Africa (SSA) [3]. It was reported 779 cases of respiratory infections per 100,000 population in 2017, and 1028 million cases of diarrhea every year in childhood [4] in sub-Saharan Africa. In addition, 24 million children were estimated to be infected by malaria in SSA in 2018 [5]. SSA, where 50% of the global under-five deaths occurred in 2018, still ranks last, with a mortality rate 78 per 1000 live births slightly above the global estimates two decades ago. The U5MR in Sierra Leone is 105 per 1000 live births, which is 1.4 times higher than the SSA estimate of U5MR [2].

To reduce the infectious disease burden, the World Health Organization (WHO) recommends integrated management of childhood illnesses (IMCI), with a strategy that includes three main elements: capacity building of health personnel, improvement of family and community practices and strengthening of health systems [6,7]. Non-government organizations contributed to the implementation of the IMCI, which is a strategy for reducing mortality and morbidity associated with major causes of childhood illnesses with the Government of Sierra Leone reinforcing institutional capacity for the treatment of acute malnutrition, prevention of malnutrition and national sensitization for nutrition security [8]. This also contributes to the early diagnosis and management of febrile children under-five [9]. In addition, for respiratory and diarrheal diseases, a series of vaccines has been recommended for children under-five years. The Expanded Program on Immunization (EPI) in Sierra Leone first included vaccines against six diseases (tuberculosis, polio, measles, diphtheria, tetanus, and pertussis), and now includes five additional diseases (yellow fever, rotavirus, hepatitis B, Haemophilus influenzae type b, and pneumococcal disease) [10]. Other measures, including improving access to safe drinking water, using improved sanitation, and improving personal and food hygiene, were part of African countries’ programs to address these diseases. For malaria, there is vector control using insecticide-treated mosquito nets (ITNs), indoor home spraying, and the prevention of infection using intermittent preventive treatment in pregnancy (IPTp) and seasonal malaria chemoprevention (SMC) in children aged 3 to 59 months [5]. These interventions contribute to reducing the burden of infectious diseases. For more efficiency in reducing the morbidity and mortality of children under-five years, earlier care-seeking at an appropriate place (with trainee health staff) with a conventional treatment is necessary. Despite these control and prevention measures implemented in most SSA countries, Sustainable Development Goal 3.2, aiming to reduce the U5MR to 25 per 1000 live births by 2030, is far from being achieved [11].

Another strategy to reduce the burden of infection is to increase care-seeking for febrile children through universal health coverage (UHC) as part of the sustainable goals aiming to make essential healthcare services available for all by reducing out-of-pocket expenses. However, UHC covers only 50% of the world population [12]. User fees can account for up to 30% of household income [13]; as a means to finance the health system, it has been shown to significantly worsen the financial hardship of households in low- and middle-income countries (LMICs) and reduce access to healthcare services. Other barriers that reduce care-seeking have also been reported. These include health facility deterrents, such as distance to the facility, sociocultural and gender factors, lack of knowledge and information about healthcare, recurrent supply stock-outs, and perceived negative behavior of health workers [14]. In Nigeria, it was reported that access to health facilities was limited due to poor road conditions in some areas. In Kenya, it was reported that mothers perceived a need to be empowered to be able to seek care for their children due to gender-related barriers. A lack of knowledge about the causes of childhood illnesses and how to prevent them, prolonged wait times, and poor communication between staff and patients were also reported to be barriers to healthcare seeking in Africa and Asia [15,16].

The commitment of African countries to implement UHC led to the FHCI for vulnerable subsets of the population [12]. Sierra Leone implemented free healthcare for pregnant women, lactating women, and children under the age of five in April 2010, covering essential care. By lowering the cost barrier [17], this policy aimed to encourage earlier healthcare seeking and management of illness in children and to reduce the mortality and morbidity rates in children under-five years. In Sierra Leone, before the FHCI, in 2007, the U5MR was 183 per 1000 live births. After the FHCI, in 2020, the U5MR was 102 per 1000 live births [18]. In addition, access to healthcare in Sierra Leone has been problematic over the past two decades, especially for vulnerable populations, due to health system constraints as a result civil wars (1991–2002) [19] and waves of disease epidemics (cholera in 2012 and Ebola in 2014–2015) [20,21].

Although the FHCI was launched 10 years ago, there are limited data on the patterns of care-seeking and no published reports describing factors associated with care-seeking behavior in Sierra Leone, even when it is known that care and treatment of the main infectious diseases are free. Such knowledge is crucial in refining public health interventions and therefore urgently needed. Using fever as an indicator of morbidity in children under-five, this study aimed to evaluate the trends in the prevalence of care-seeking for fever and to identify the determinants of healthcare service utilization by caregivers of children under-five years of age in the context of FHCI and the Ebola emergency.

## Methods

### Data source

This study used data from the Sierra Leone Demographic and Health Surveys (DHS) of 2008, 2013, and 2019 and the Malaria Indicator Survey (MIS) of 2016. These were nationally representative household surveys in which women aged 15 to 49 years were interviewed. The surveys used 3 questionnaires, a household questionnaire, a women’s (15–49 years old) questionnaire and a men’s (15–59 years old) questionnaire. Our study concerned children under the age of 5 years who had febrile episodes in the two last weeks preceding each survey in Sierra Leone.

### Settings

Sierra Leone is a country in West Africa with an area of 71,740 km^2^ and an estimated population in 2016 of 7,396,000 inhabitants [22]. The climate is tropical with vegetation ranging from savannah to forest. Sierra Leone is divided into 4 administrative regions: northern, eastern, southern, and western. The northern region was divided into North and North-west in 2019. These regions are subdivided into 14 districts, and into 16 from 2017. The health system is organized into 3 levels: The first is primary health care (PHC), with peripheral health units (PHUs): 233 community health centers (CHCs), 319 community health posts (CHPs), and 632 maternal and child health posts (MCHPs). The secondary level is made up of 21district hospitals [23]. The tertiary level includes regional and specialized hospitals. There are 6 hospitals in a teaching hospital complex established by an act of Parliament. There are several private clinics and hospitals spread across the 14 districts of the country. Wealth is not distributed equally within rural and urban areas. About 61% and 1.4% of the population are the richest respectively in the urban and in the rural areas, and about 3% and 28% of the population are the poorest respectively in the urban and in the rural areas [24].

### Variables

The outcome variable was the proportion of children under-five years of age whose caregivers had sought care during a feverish illness in the 2 weeks preceding the survey.

Independent variables included those describing sociodemographic data and those assessing the determinants of care-seeking for fever: respondent’s age (15−24, 25−34, and ≥35 years); level of education of the child’s mother (no formal education, primary, secondary, or higher level education); number of children ever born in the household (1−2, 3−4, and 5≥ children); the sex of the head of the child’s household; the age of the head of the household (15−24, 25–34, and ≥35 years); region (eastern, northern, southern, western, and northwestern in 2019); place of residence (urban or rural); religion (Christian, Muslim, traditional, or other); the sex of the child; the age of the child (<12, 12−35, and 36–59 months); and the place where health care is sought (public, private, traditional, or other). The wealth quintile (richest, richer, middle, poorer, and poorest) was used; its construction was based on survey data about the household’s ownership of consumer goods, dwelling characteristics, drinking water source, toilet facilities, and other characteristics that relate to a household’s socioeconomic status. The resulting combined wealth index has a mean of 0 and a standard deviation of 1. Once the index was computed, national-level wealth quintiles (from lowest to highest) were obtained by assigning household scores to each de jure household member, ranking each person in the population by their score, and then dividing the ranking into 5 equal categories, each comprising 20% of the population [24].

### Sampling method

The four Sierra Leone population-based surveys used a 2-stage cluster sampling method. Enumeration areas (EAs) were constructed with complete coverage of the country. Each EA included several households. At the first stage, EAs were selected with stratified probability proportional to sample size. The place of residence (urban or rural) was used to stratify EAs. At the secondary stage, households were selected from the EAs using systematic random sampling. The frames were developed based on the 2004 census for the 2008 and 2013 surveys and the 2015 census for the 2016 and 2019 surveys [24–26].

### Statistical methods

Statistical analyses were performed using Stata version 15.0. The northern region was divided into North and North-west in 2019, for the comparison purpose with the 2008, 2013, and 2016 data, these 2 regions were combined during data analysis. We first described the characteristics of parents of children and those of children with fever during the two weeks preceding the survey. Chi-square test was used to assess differences between participants’ characteristics over the surveys. The descriptive analyses were weighted for probability sampling and considering stratification and clustering, as is standard in all DHS program surveys [24,25,27]. We compared the percentages of care-seeking between the four surveys adjusting for the participants’ characteristics using a multivariable modified Poisson regression model with generalized estimating equations. We also performed a modified Poisson regression model using a generalized estimating equations to identify the determinants of care-seeking under the FHCI (2013–2019). A two-sided P-value of 0.05 or less was considered to indicate statistical significance.

### Ethics

The Sierra Leone National Ethics Committee and the International Review Board of International Coach Federation (ICF) approved the use of the surveys, and the participants’ written consent was obtained before data collection. We were authorized by the Demographic and Health Survey program to access data at https://dhsprogram.com/data.

## Results

### Characteristics of the study population

Overall, 1208 children under -five years of age with fever were included in the 2008 survey, 2823 in the 2013 survey, 1633 in the 2016 survey, and 1464 in the 2019 survey.

In the four surveys, most (more than 45%) of the women were 25 to 34 years old. The majority of caregivers were women (more than 52%) who had no level of education across the surveys. The percentage of caregivers’ woman who were Muslim was about 75%, and more than 65% of the women lived in rural areas. At least 40% were in the poor and poorest groups across the four surveys. For more than 75% of households, the head was a man over 35 years old. The percentage of children aged between 12 and 35 months old was at least 45% across the surveys. About 38% of children lived in the northern region, except in 2019. There was no statistically significant difference between sex of the household head, age of the household head, distribution of wealth, regions, urban and rural areas or between sex of the child for the four surveys (Table 1).

**Table 1:** Characteristics of the children’s caregivers and children with fever in the four surveys.

Characteristics of the population	2008		2013		2016		2019		p-value
	N	%	N	%	N	%	N	%	
**PARENTS**	** **	** **	** **	** **	** **	** **	** **	** **	** **
**Age of mother (years)**	1208		2823		1633		1464		0.03
15–24		28.7		27.1		33.5		28.0	
25–34		47.3		47.7		45.6		47.5	
≥35		24.0		25.2		20.9		24.5	
**Number of children**	1208		2823		1633		1464		0.04
1–2		35.7		31.8		31.6		36.3	
3–4		30.8		31.4		31.9		32.6	
≥5		33.5		36.8		36.5		31.1	
**Mother’s education level**	1208		2823		1633		1464		<0.001
None		72.5		68.3		59.4		52.5	
Primary		13.9		14.7		15.1		18.1	
Secondary or higher		13.6		17.0		25.5		29.4	
**Sex of the household head**	1208		2823		1633		1464		0.06
Male		81.4		75.7		77.5		76.8	
Female		18.6		24.3		22.5		23.2	
**Age of the household head(years)**	1208		2823		1633		1464		0.76
15–24		2.8		3.8		3.2		3.6	
25–34		22.9		23.7		21.5		22.6	
≥35		74.3		72.4		75.2		73.8	
**Wealth index**	1208		2823		1633		1464		0.95
Poorest		21.4		22.8		22.8		24.7	
Poorer		21.4		21.1		23.0		21.2	
Middle		21.2		20.4		21.8		20.6	
Richer		18.8		20.0		19.0		19.1	
Richest		17.2		15.6		13.4		14.4	
**Region**	1208		2823		1633		1464		0.26
Eastern		18.1		22.1		24.5		26.0	
Northern		45.1		41.7		37.8		31.5	
Southern		20.2		23.5		24.9		24.2	
Western		16.6		12.7		12.8		18.3	
**Place residence**	1208		2823		1633		1464		0.07
Urban		27.5		27.1		35.1		34.7	
Rural		72.5		72.9		64.9		65.3	
**Religion**	1208		2823		1633		1464		<0.001
Muslim		79.5		84.3		74.6		81.2	
Christians		19.2		15.4		25.2		18.8	
Traditional/others		1.3		0.4		0.2		0	
**CHILDREN**									
**Sex of the child**	1208		2823		1633		1464		0.75
Male		51.4		49.4		50.1		50.1	
Female		48.6		50.6		49.9		49.9	
**Age (months)**	577		1371		1633		786		<0.001
< 12		27.8		21.7		20.5		20.0	
12–35		48.2		45.7		45.1		48.0	
36–59		23.9		32.5		34.4		32.0	

### Trends of the prevalence of health care-seeking for fever

**Fig 1:**
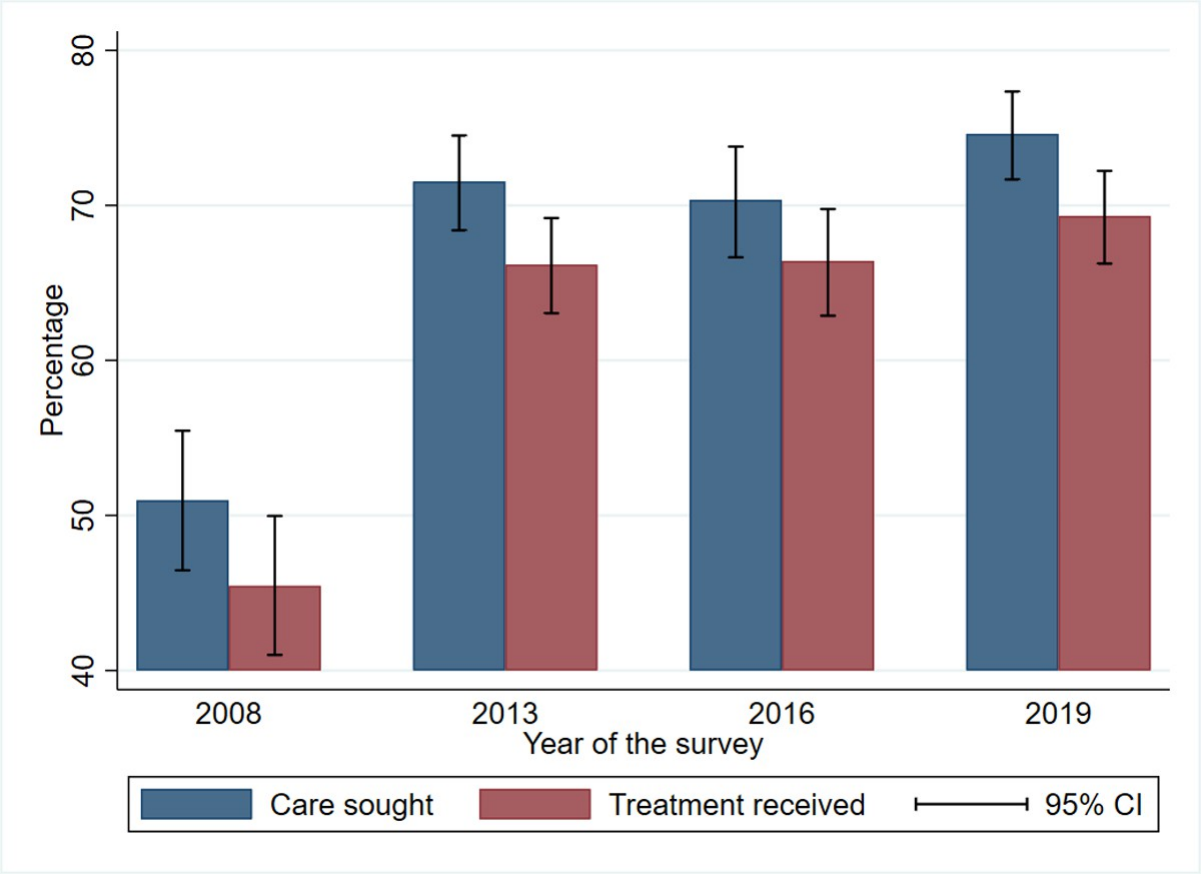
Care sought and treatment received by children under-five by survey.

The trends of care-seeking for fever in children under-five years of age in the 2 weeks preceding each survey on the Fig 1, shows an increase from 51% (95% CI (46.4−55.5)) in 2008 to 71.5% (95% CI (68.4−74. 5)) in 2013, but a decrease to 70.5% (95% CI (66.6−73.8)) in 2016 relative to 2013. An increase to 74.6% (95% CI (71.6−77.3)) in 2019 was seen. Treatment of febrile children increased from 45.4% (95% CI (41.0−49.9)) in 2008 to 69.3% (95% CI (66.2−72.2)) in 2019.

**Fig 2:**
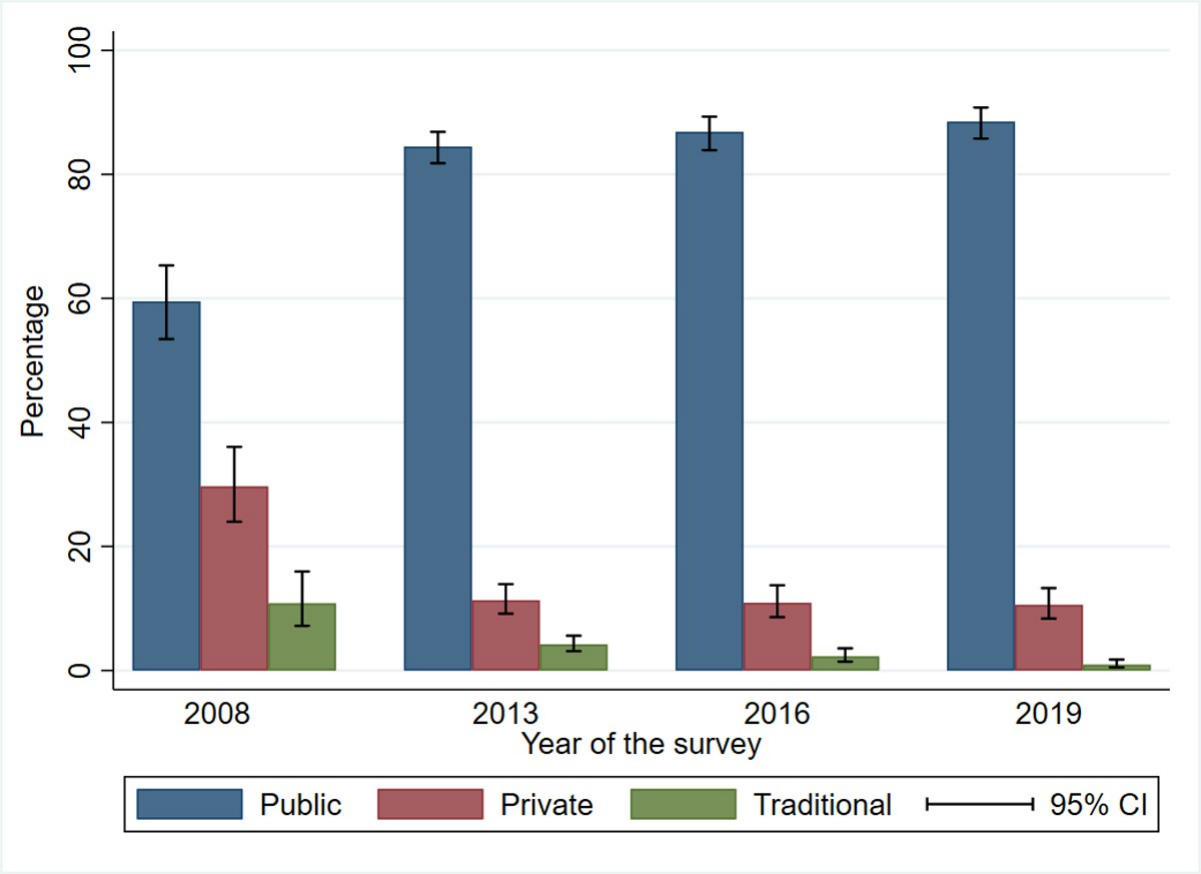
Place of care sought by caregivers of children under-five by survey.

Care-seeking at public health centers increased from 59.5% (95% CI (53.4−65.3)) in 2008 to 84.5% (95% CI (81.8−86.9)) in 2013, and to 86.8% (95% CI (83.9−89.3)) in 2016. In 2019, care-seeking at public health centers was 88.5% (95% CI (85.7−90.7). Healthcare seeking in the private sector decreased from 29.6% (95% CI (23.9−36.0)) in 2008 to 10.6% (95% CI (8.3−13.2)) in 2019, and the proportion of traditional treatment decreased from 10.8% in 2008 to less than 1% in 2019 (Fig 2).

**Fig 3:**
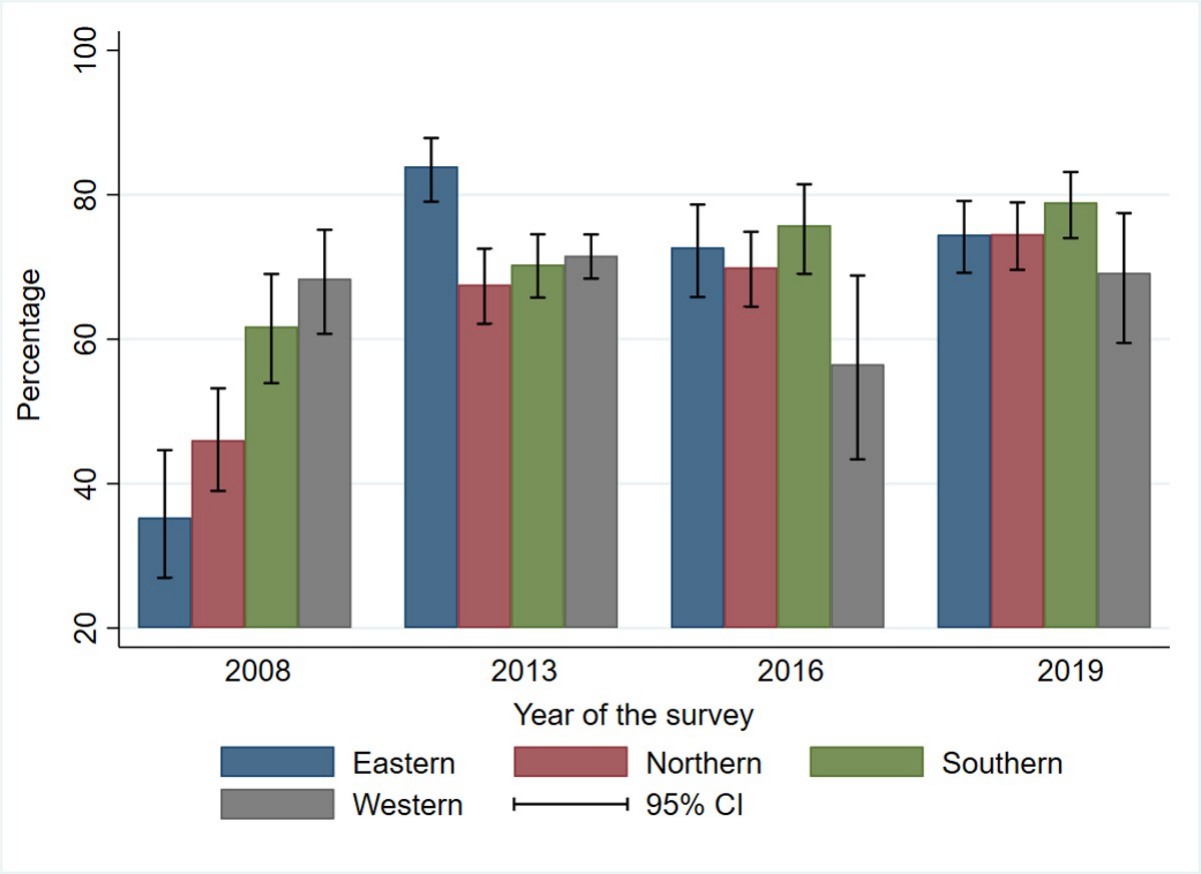
Care sought by caregivers of children under-five in the regions by survey.

In 2008, the level of care-seeking for fever was high in the western and southern regions at 68.4% (95% CI (60.7−75.1)) and 61.8% (95% CI (53.9–69.8)), respectively. A decrease in care-seeking for fever was noted in the western region, from 68.4% (95% CI (60.7−75.1)) in 2008 to 56.6% (95% CI (43.4−68.8)) in 2016, but there was an increase to 69.2% (95% CI (59.4−77.4)) in 2019 (Fig 3).

Care-seeking prevalence was 1.5 times higher in 2013 than in 2008 (prevalence ratio,1.48; 95% CI (1.33−1.65); p < 0.001), 1.4 times higher in 2016 than in 2008 (prevalence ratio, 1.43; 95% CI (1.28−1.59); p < 0.001), and 1.5 times higher (prevalence ratio, 1.51; 95% CI (1.36−1.68); p < 0.001) after adjusting for mother’s age and, education level, the sex of the household head, religion, and the child’s age (Table 2).

**Table 2:** Comparing the prevalence of care-seeking between the four surveys prevalence adjusted for characteristics of caregivers and children.

Characteristics	Total No of children with fever	No of children who sought care			
			Adjusted PR*	95% CI	p-value
**Year of the survey**					<0.001** **
2008	1208	630	1		
2013	2823	2080	1.48	(1.33–1.64)	
2016	1633	1173	1.43	(1.29–1.59)	
2019	1464	1121	1.51	(1.36–1.68)	
**PARENTS**					
**Age of mother (years)**					0.009
15–24	2122	1535	1		
25–34	3359	2379	1.02	(0.97–1.07)	
≥35	1647	1090	0.93	(0.88–0.99)	
**Mother’s education level**					<0.001
Secondary or high	1502	1163	1		
None	4521	3041	0.90	(0.85–0.94)	
Primary	1105	800	0.91	(0.86–0.97)	
**Sex of the household head**					0.11
Female	1629	1200	1		
Male	5499	3804	0.96	(0.91–1.00)	
**Religion**					0.74
Christians	1405	988	1		
Muslim	5698	4005	1.02	(0.96–1.07)	
Traditional/others	25	11	0.96	(0.67–1.39)	
**CHILDREN**					
**Age of the child (months)**					<0.001
< 12	950	714	1		
12–35	2024	1442	0.93	(0.89–0.98)	
36–59	1393	923	0.87	(0.82–0.91)	

*Prevalence Ratio.

### Determinants of care-seeking for fever under the free-of-charge policy

The results presented in Table 3 indicate that the age of the child’s mother, the socioeconomic status of the household, the region and the age of the child were significantly associated with care-seeking. The level of care-seeking was highest when the mother was younger than 35 years old and when the family was from the eastern region or the child was younger than 12 months. A trend was observed for the sex and age of the household head. The level of care-seeking was lowest when the household head was a man and was 15−24 years old.

**Table 3:** Multivariable modified Poisson regression model to identify factors associated with care-seeking among children under-five with fever under the free-of-charge policy, 2013–2019.

Characteristics	Total No of children with fever	No of children who sought care	Non-adjusted PR*	95% CI	Adjusted PR*	95% CI	p-value for adjusted model
**PARENTS**							
**Age of mother (years)**							0.03
15–24	1759	1344	1		1		
25–34	2772	2061	0.97	(0.93–1.00)	1.01	(0.96–1.06)	
≥35	1389	969	0.91	(0.86–0.95)	0.93	(0.85–1.01)	
**Mother’s education level**							0.15
Secondary or high	1314	1030	1		1		
None	3691	2652	0.91	(0.88–0.95)	0.94	(0.90–1.00)	
Primary	915	692	0.96	(0.91–1.00)	0.97	(0.90–1.03)	
**Number of children**							0.51
1–2	1955	1494	1		1		
3–4	1864	1388	0.97	(0.93–1.01)	0.96	(0.91–1.02)	
≥5	2101	1492	0.92	(0.88–0.96)	0.98	(0.92–1.05)	
**Sex of the household head**							0.07
Female	1393	1062	1		1		
Male	4527	3312	0.96	(0.93–1.00)	0.95	(0.91–1.00)	
**Age of the household head (years)**							0.10
15–24	211	152	1		1		
25–34	1369	1033	1.05	(0.95–1.15)	1.15	(1.01–1.31)	
≥35	4340	3189	1.02	(0.93–1.11)	1.14	(1.00–1.30)	
**Wealth index**							<0.001
Poorest	1494	1036	1		1		
Poor	1304	952	1.05	(1.00–1.10)	1.09	(1.03–1.16)	
Middle	1251	958	1.11	(1.06–1.17)	1.14	(1.07–1.22)	
Richer	1167	911	1.11	(1.05–1.18)	1.15	(1.05–1.23)	
Richest	704	517	1.05	(0.99–1.12)	1.13	(1.03–1.25)	
**Region**							<0.001
Eastern	1264	994	1	1	1		
Northern	2409	1732	0.91	(0.86–0.96)	0.90	(0.85–0.96)	
Southern	1659	1256	0.96	(0.91–1.01)	0.99	(0.93–1.06)	
Western	588	392	0.87	(0.81–0.94)	0.82	(0.73–0.91)	
**Place residence**							0.42
Urban	1743	1340	1		1		
Rural	4177	3034	0.94	(0.90–0.98)	0.97	(0.91–1.03)	
**Religion**							
Christians	1127	842	1				
Muslim	4783	3524	0.98	(0.94–1.03)			
Traditional/others	10	8	1.10	(0.72–1.66)			
**CHILDREN**							
**Gender of the child**							
Female	2941		1				
Male	2160		1.01	(0.98–1.04)			
**Age of the child (months)**							<0.001
< 12	795	626	1		1		
12–35	1744	1313	0.95	(0.91–0.99)	0.95	(0.90–0.99)	
36–59	1251	856	0.86	(0.82–0.91)	0.87	(0.82–0.92)	

*Prevalence Ratio.

## Discussion

Using nationally representative health data on children under-five living in Sierra Leone, we have observed that the implementation of the FHCI in 2010 coincided with an increase of more than 20 percentage points in the prevalence of care-seeking for febrile children in 2019 compared to 2008, and most of the children received treatment in the public health sector. Traditional treatment was significantly reduced in 2019 compared to 2008. In addition, we identified several determinants of care-seeking behavior related to children and caregivers, among which the age of the mother, the region of residence, the household socioeconomic status, and the age of the child were the most significant.

We have observed in our study that caregivers of children under-five sought more healthcare for fever after the FHCI was implemented than before the FHCI. The rise we observed in the prevalence of care-seeking for febrile children following the implementation of the FHCI in Sierra Leone is in keeping with the findings in Madagascar, and in Kenya, where similar increases in care-seeking for children under-five years of age after the initiation of free health care were also reported [28,29].

In the present study, considering the period following the implementation of the FHCI, the prevalence of care-seeking for febrile children was lower for households in the poorest quintile. The limited ability of the poorest household to bear the indirect costs of healthcare (loss of wages, loss of productivity, etc.) likely contributes to this difference. In addition, recurrent drug and supply stock-outs and the resulting out-of-pocket expenses incurred for health services despite the FHCI [13], contribute to the difference between wealth quintiles in terms of care-seeking. A similar finding was reported in a previous study in Sierra Leone (2008–2013) among mothers and their child about institutional delivery, antenatal care and postnatal care, which showed an increase in the prevalence of care-seeking after the abolition of user fees left the most financially vulnerable people behind [17].

We also found a lower prevalence of care-seeking for fever in children over one year of age. The same association was found in other studies in Africa [30,31]. This may be explained by the fact that caregivers of older children, having already dealt with several episodes of febrile illnesses during the child’s life, may have become experienced in treating fevers without relying on a public health center.

In addition, the northern and western regions had a lower prevalence of care-seeking compared to the eastern region. The population-based surveys in 2013 and 2016 coincided with periods of outbreaks in Sierra Leone, the cholera outbreak from 2012 and the Ebola outbreak from 2014–2015. During these outbreaks, the northern and western regions were the most affected [20,21], and the population in these regions may have avoided health centers for fear of contamination. This could explain the low prevalence of care-seeking for children under-five in the context of free health care observed in our study, especially since an increase in care-seeking in 2019 was observed. In addition, poor road conditions, the lack of availability of affordable and reliable transport, and physical inaccessibility in the rainy-season may also explain the persistent low prevalence of care-seeking in these regions despite the FHCI [14].

Another finding was a higher prevalence of care-seeking when the mother’s of the child was older than 25 years compared to one who was younger than 25 years or when the household head was a woman. A possible explanation could be the limited experience of young caregivers with the manifestations of childhood diseases, as reported in studies conducted in Sierra Leone and Nigeria, where the failure to recognize symptoms of childhood febrile illnesses led to delays in seeking care or not seeking care at all [32,33]. These results also highlight the gender influence at the level of household decisions on seeking care for sick children. Improved care-seeking behavior by caregivers of children in Ghana, Kenya, and Zambia was reported when both parents were involved in the decision [34].We acknowledge some limitations in our study. The data collection might have been subject to social desirability bias. On the other hand, we cannot exclude that health system interventions such as IMCI, NGOs, the attitudes of health workers, and other interventions might contribute to the increased health care-seeking for children under-five [13]. Additionally, some aspects, such as the social networks of the caregivers, the perception of the need for healthcare services, and the causes of childhood illnesses, which contribute to care-seeking behavior could be better addressed by qualitative research, which was not included in the population-based surveys we used as data for our analyses.

## Conclusion

Our study reveals that care-seeking for children under-five increased significantly after the implementation of the FHCI in the public health sector in Sierra Leone. Seeking care for fever varied by the age of the mother, the age of the child, the region, and household wealth. Maintaining the FHCI with adequate strategies to address other barriers beyond financial ones is critical.
